# Supplementary material for: Unraveling the mechanism for paired electrocatalysis of organics with water as a feedstock
Source: Nat Commun. 2022 Jun 6;13:3125. doi: 10.1038/s41467-022-30495-1 (PMC9170728; doi:10.1038/s41467-022-30495-1)
Supplement: Supplementary file 1 — Supplementary Information [file 41467_2022_30495_MOESM1_ESM.pdf]

Supplementary Information

Supplementary Information for:

Unraveling the mechanism for paired electrocatalysis of organics with water as a feedstock

Ganceng Yang, Yanqing Jiao, Haijing Yan,* Ying Xie, Chungui Tian, Aiping Wu, Yu Wang & Honggang Fu*

Key Laboratory of Functional Inorganic Material Chemistry Ministry of Education of the People's Republic of China, Heilongjiang University, Harbin, China.

E-mail: yanhaijing@hlju.edu.cn; fuhg@hlju.edu.cn; fuhg@vip.sina.com

Contents

1. Supplementary Figures 4
Supplementary Fig. 1 4
Supplementary Fig. 2 5
Supplementary Fig. 3 6
Supplementary Fig. 4 7
Supplementary Fig. 5 8
Supplementary Fig. 6 9
Supplementary Fig. 7 10
Supplementary Fig. 8 11
Supplementary Fig. 9 12
Supplementary Fig. 10 13
Supplementary Fig. 11 14
Supplementary Fig. 12 15
Supplementary Fig. 13 16
Supplementary Fig. 14 17
Supplementary Fig. 15 18
Supplementary Fig. 16 19
Supplementary Fig. 17 20
Supplementary Fig. 18 21
Supplementary Fig. 19 22
Supplementary Fig. 20 23
Supplementary Fig. 21 24
Supplementary Fig. 22 25
Supplementary Fig. 23 26
Supplementary Fig. 24 27
Supplementary Fig. 25 28
Supplementary Fig. 26 29
Supplementary Fig. 27 30
Supplementary Fig. 28 31
Supplementary Fig. 29 32

Supplementary Fig. 30 33
Supplementary Fig. 31 34
Supplementary Fig. 32 35
Supplementary Fig. 33 36
Supplementary Fig. 34 37
Supplementary Fig. 35 38
Supplementary Fig. 36 39
2. Supplementary Tables 40
Supplementary Table 1 40
Supplementary Table 2 41
Supplementary Table 3 42
Supplementary Table 4 43
Supplementary Table 5 44
Supplementary Table 6 45
Supplementary Table 7 46
Supplementary Table 8 47
Supplementary Table 9 48
References 49

1. Supplementary Figures

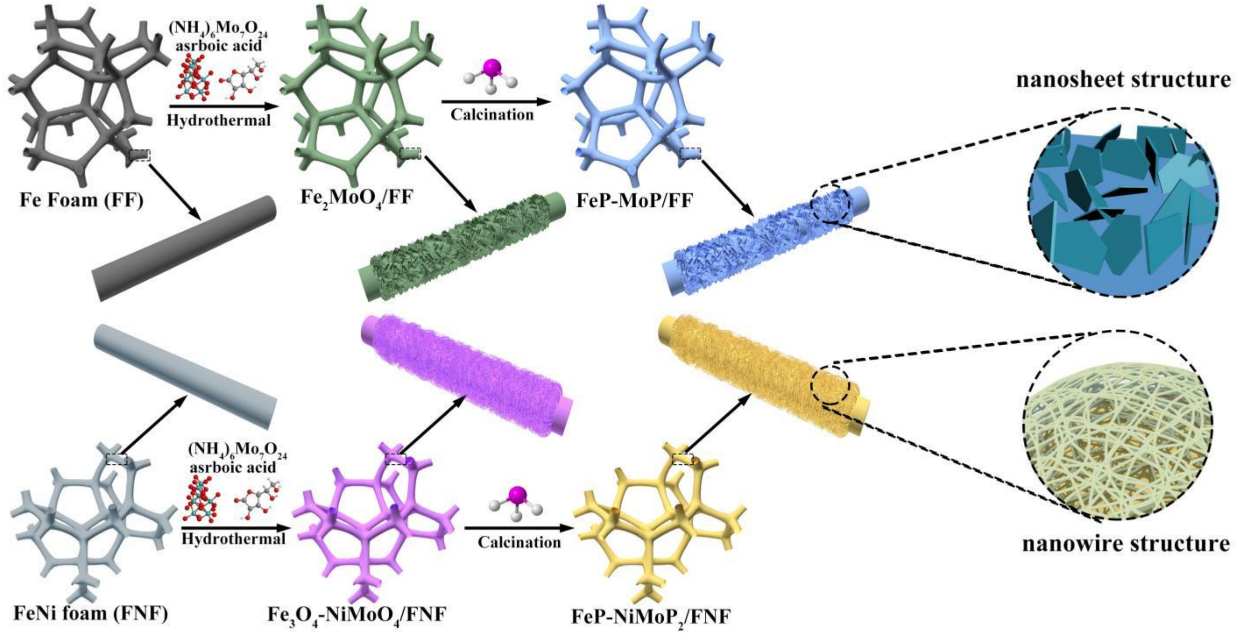

Supplementary Fig. 1 | Schematic illustration of the formation of the FeP-MoP/FF and FeP-$NiMoP_2$/FNF.

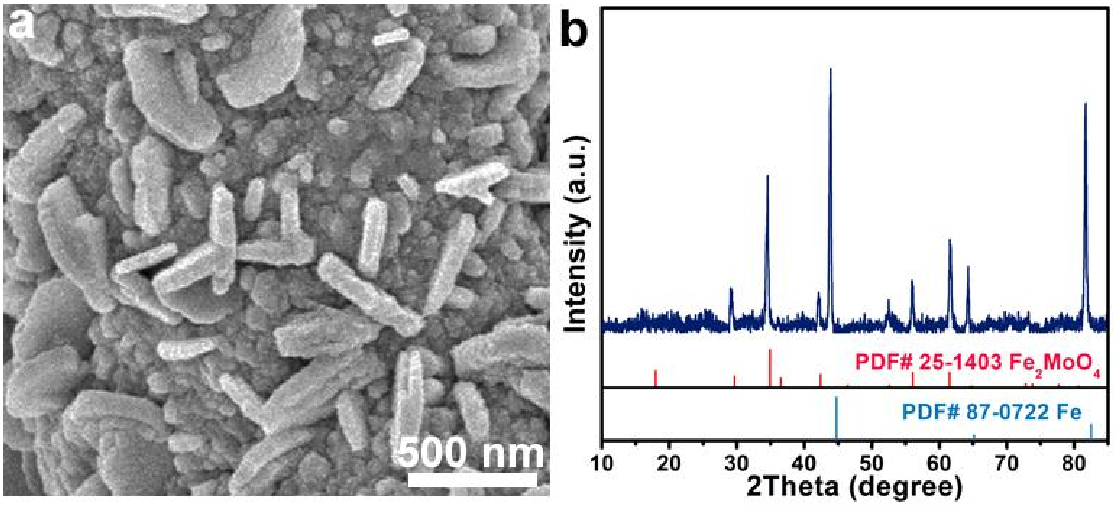

Supplementary Fig. 2 | (a) SEM image and (b) XRD pattern of Fe_2MoO_4/FF precursor hybrid.

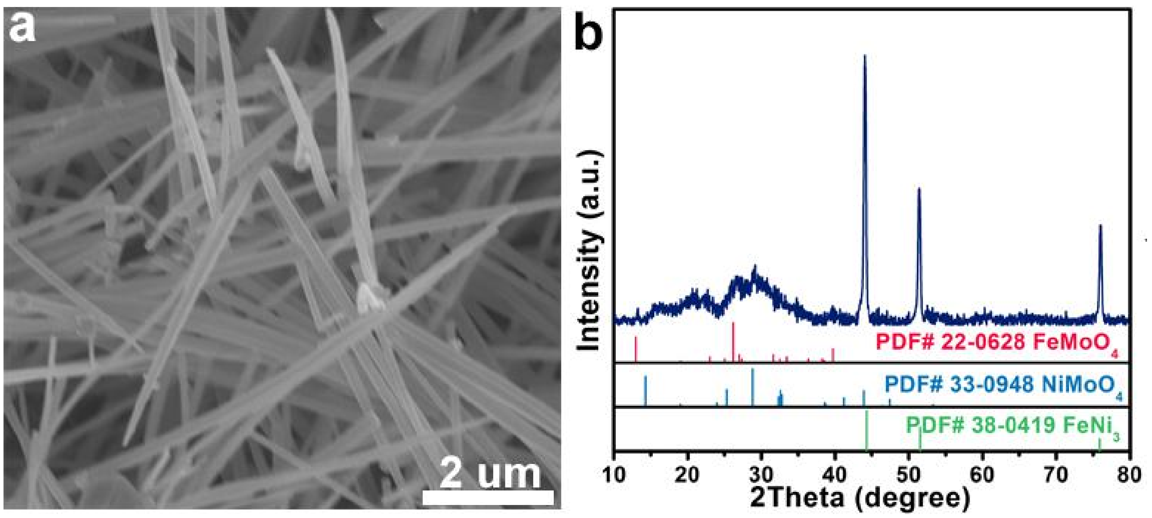

Supplementary Fig. 3 | (a) SEM image and (b) XRD pattern of $FeMoO_4$-$NiMoO_4$/FNF precursor hybrid.

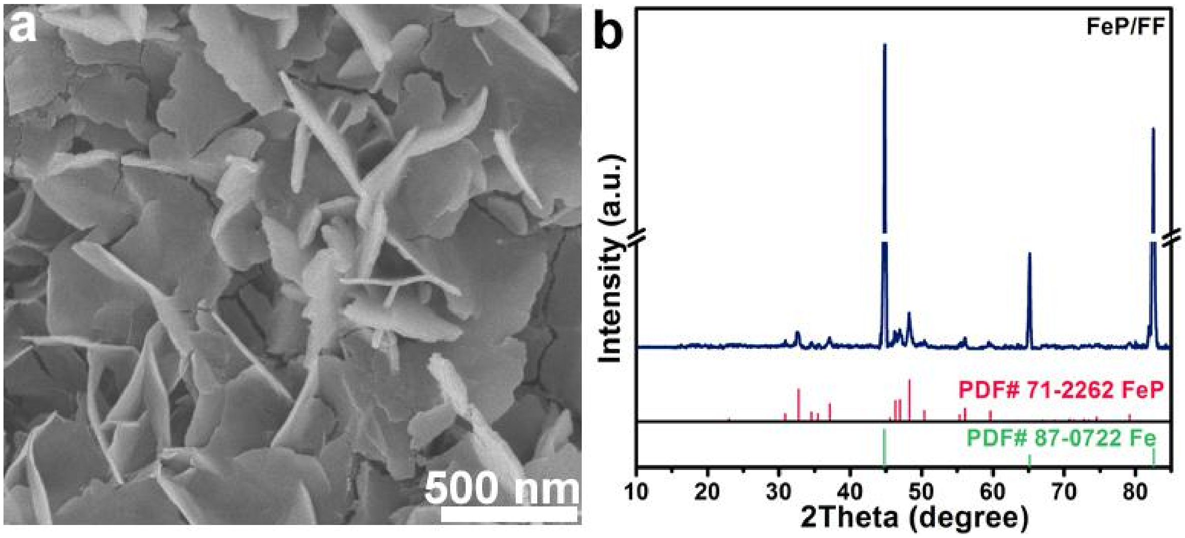

Supplementary Fig. 4 | (a) SEM image and (b) XRD pattern of FeP/FF hybrid.

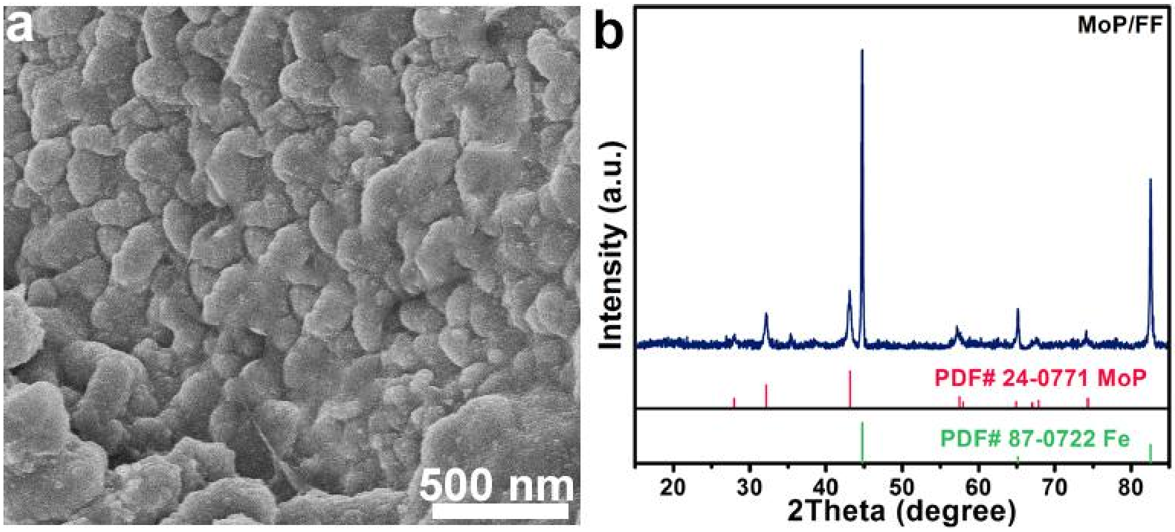

Supplementary Fig. 5 | (a) SEM image and (b) XRD pattern of MoP/FF hybrid.

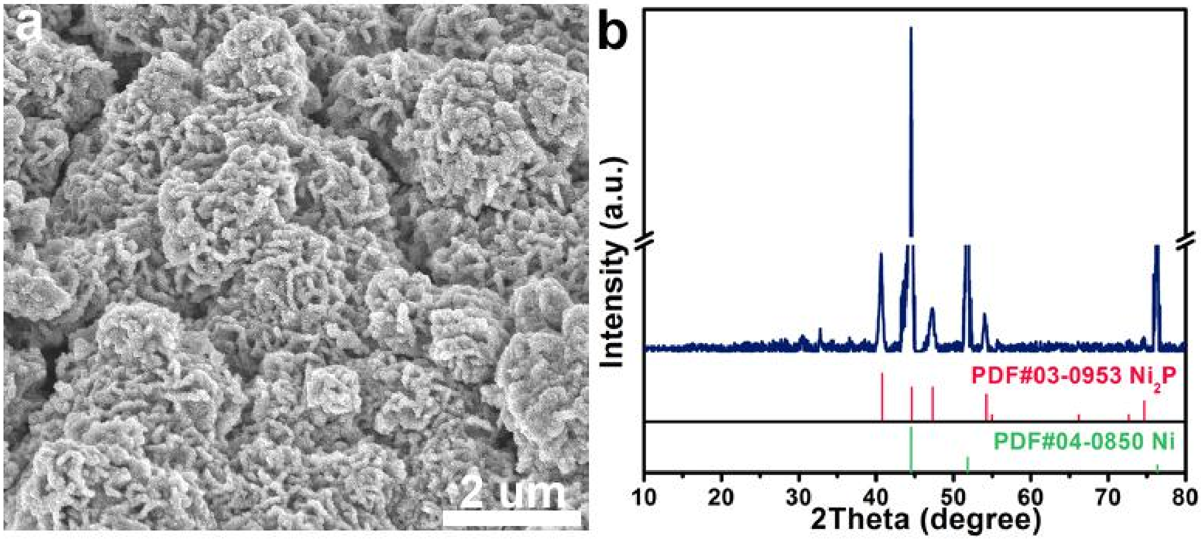

Supplementary Fig. 6 | (a) SEM image and (b) XRD pattern of Ni_2P/NF hybrid.

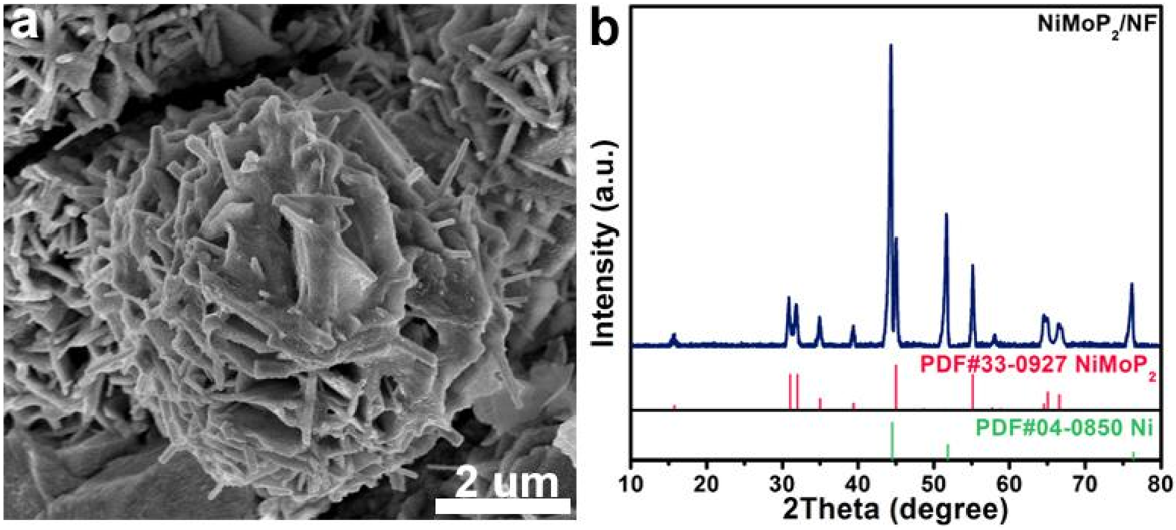

Supplementary Fig. 7 | (a) SEM image and (b) XRD pattern of $NiMoP_2$/NF hybrid.

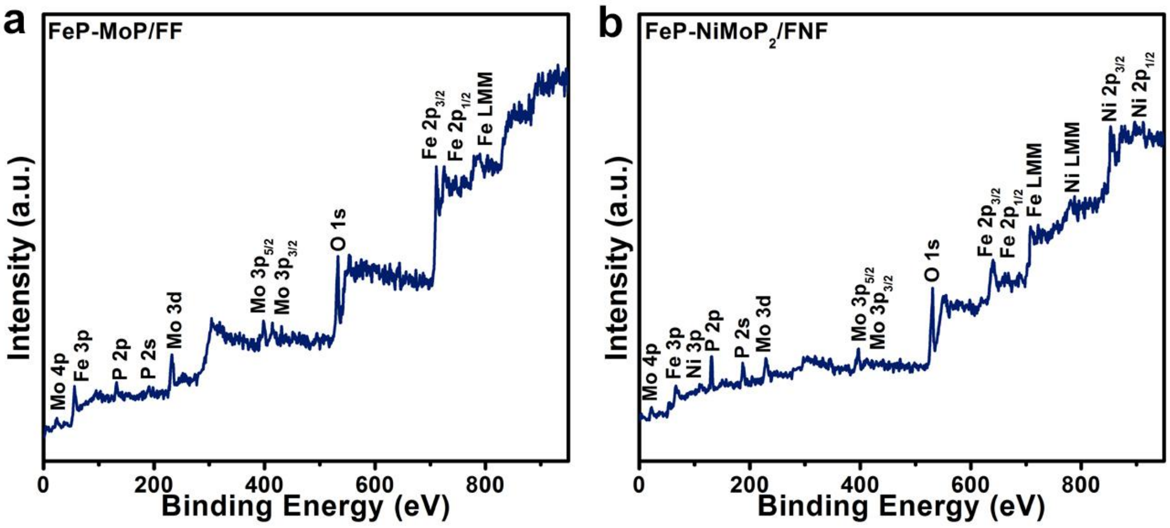

Supplementary Fig. 8 | The survey spectra of (a) FeP-MoP/FF hybrid and (b) FeP-$NiMoP_2$/FNF hybrid.

The result reveals that the FeP-MoP/FF is mainly composed of Fe, Mo and P elements and the FeP-$NiMoP_2$/FNF is mainly composed of Fe, Ni, Mo and P elements.

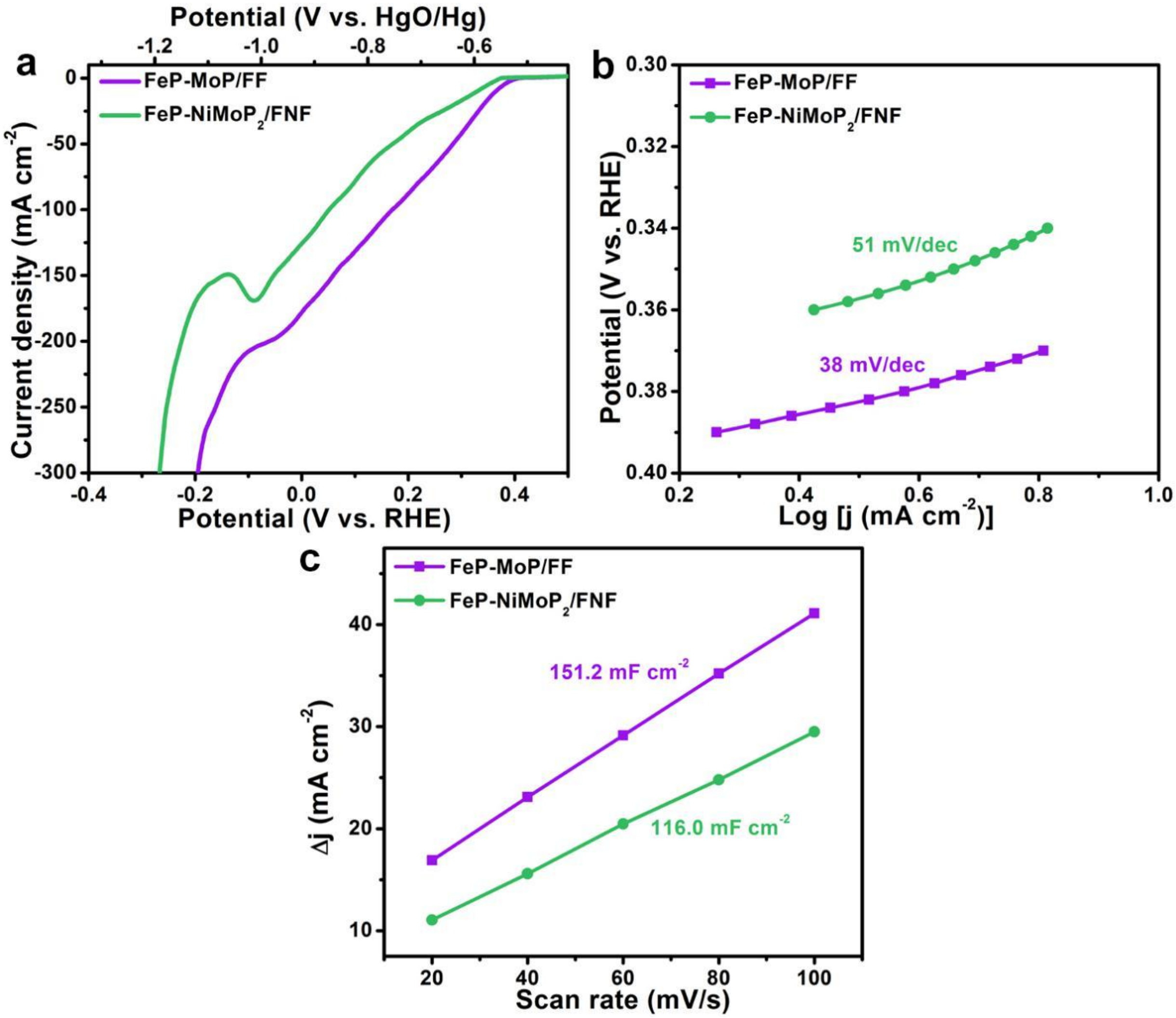

Supplementary Fig. 9 | (a) LSV curves, (b) Tafel slops and (c) capacitive curves of FeP-MoP/FF and FeP-$NiMoP_2$/FNF in 1.0 M KOH with 10 mM 4-NBA.

FeP-MoP/FF shows the higher activity and smaller Tafel slope than FeP-$NiMoP_2$/FNF for 4-NBA ERR. Meanwhile, FeP-MoP/FF shows the larger ECSA than FeP-$NiMoP_2$/FNF, also indicating the better intrinsic activity of FeP-MoP/FF than FeP-$NiMoP_2$/FNF

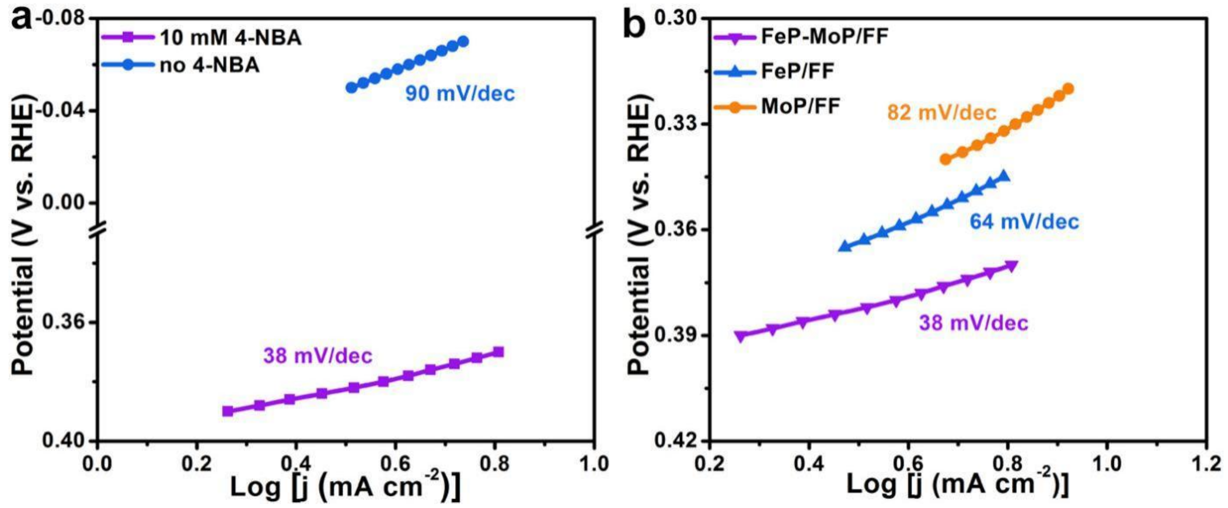

Supplementary Fig. 10 | Tafel slops of (a) FeP-MoP/FF in 1.0 M KOH without and with 10 mM 4-NBA, and (b) FeP-MoP/FF, FeP/FF and MoP/FF for 4-NBA ERR in 1.0 M KOH.

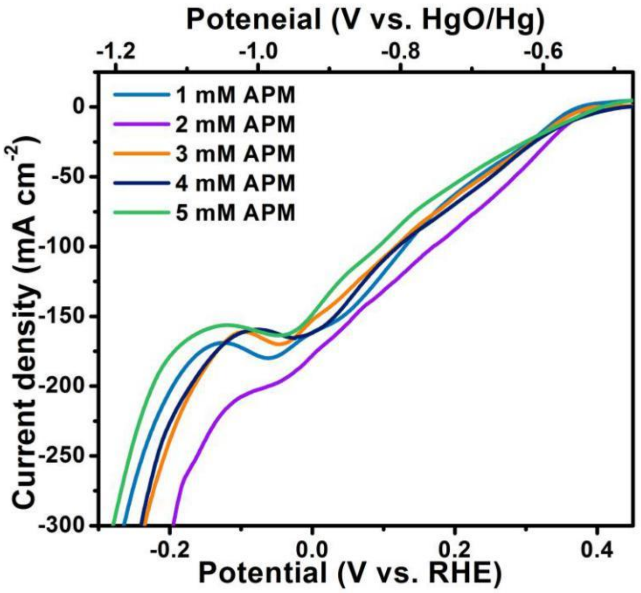

Supplementary Fig. 11 | LSV curves of synthesized FeP-MoP/FF by using different APM concentrations for 4-NBA ERR.

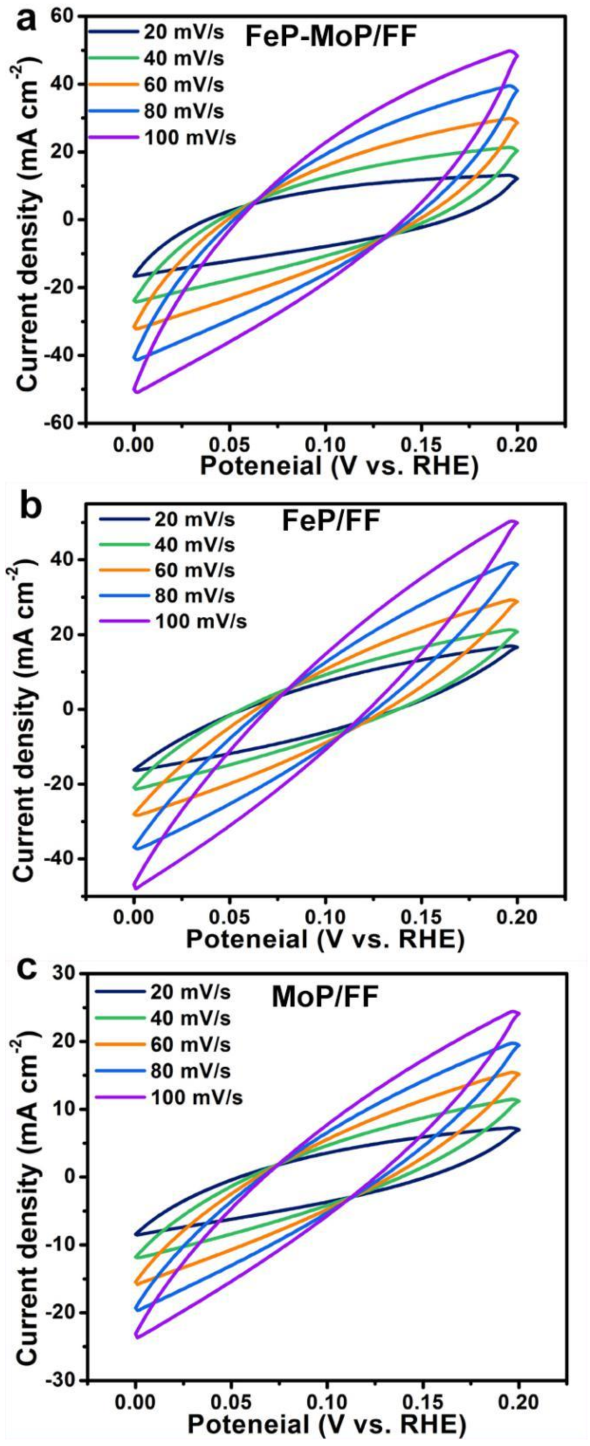

Supplementary Fig. 12 | CVs of the catalysts for cathodic reduction in the region of 0-0.2 V, (a) FeP-MoP/FF, (b) FeP/FF and (c) MoP/FF.

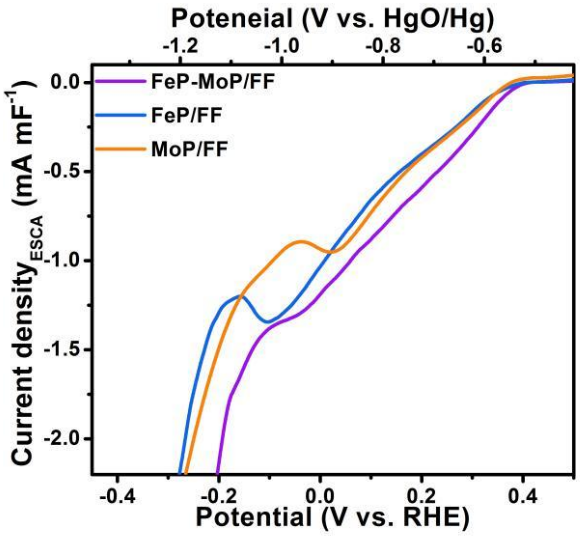

Supplementary Fig 13. (a) LSV curves with normalization by ECSA of FeP-MoP/FF, FeP/FF and MoP/FF.

We have calculated the ECSA-normalized activity by of FeP-MoP/FF, FeP/FF and MoP/FF catalysts. As shown, FeP-MoP/FF delivers higher ECSA-normalized activity compared to the FeP/FF and MoP/FF, further suggesting the excellent intrinsic 4-NBA EER activity of FeP-MoP heterojunction. Actually, apart from the high surface area, other merits, such as outstanding electron interaction and rapid electronic conductivity also contribute the enhanced activity of FeP-MoP/FF, which is the result of comprehensive effects.

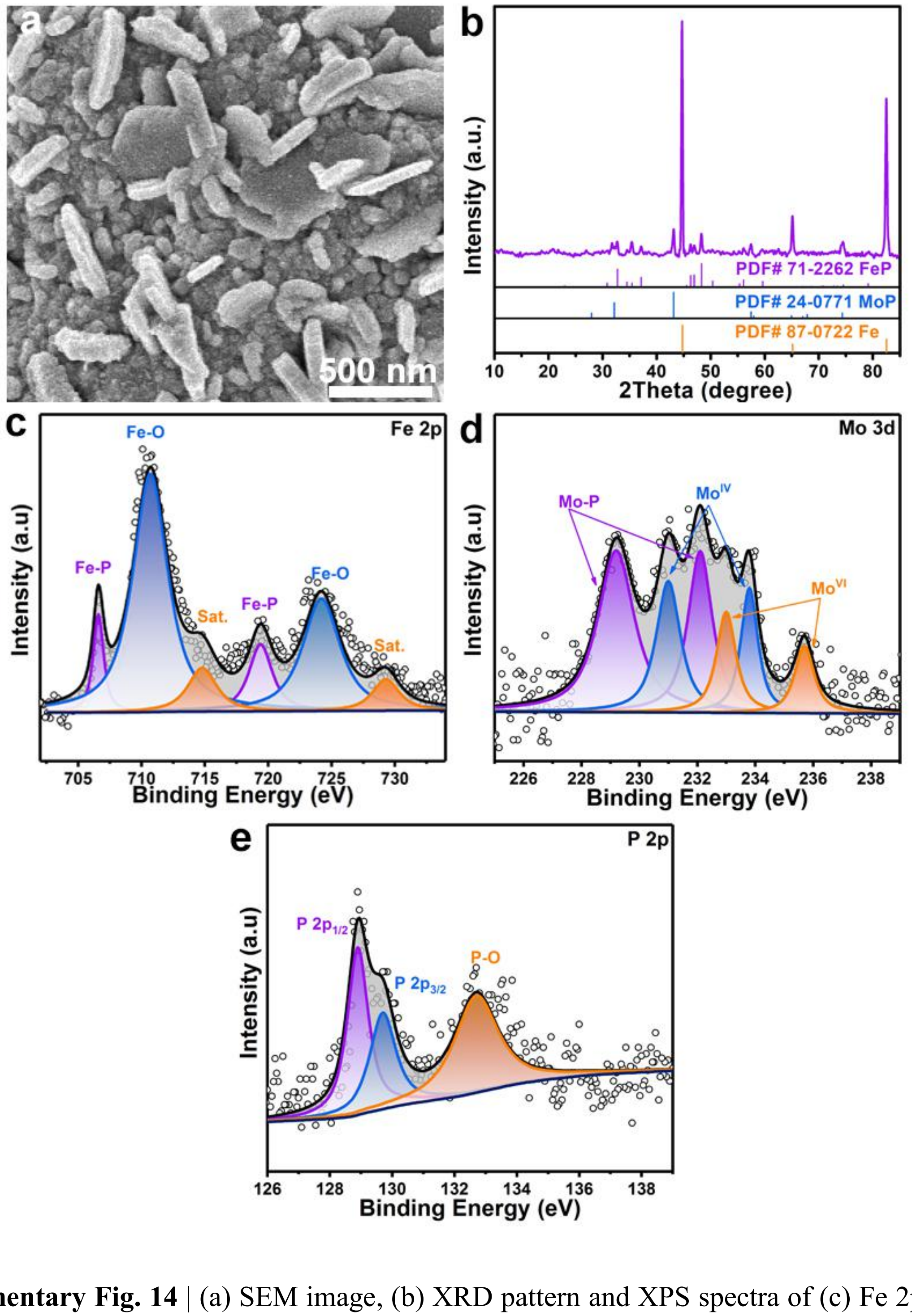

Supplementary Fig. 14 | (a) SEM image, (b) XRD pattern and XPS spectra of (c) Fe 2p, (d) Mo 3d and (e) P 2p of FeP-MoP/FF hybrid after 4-NBA ERR for ten successful cycles.

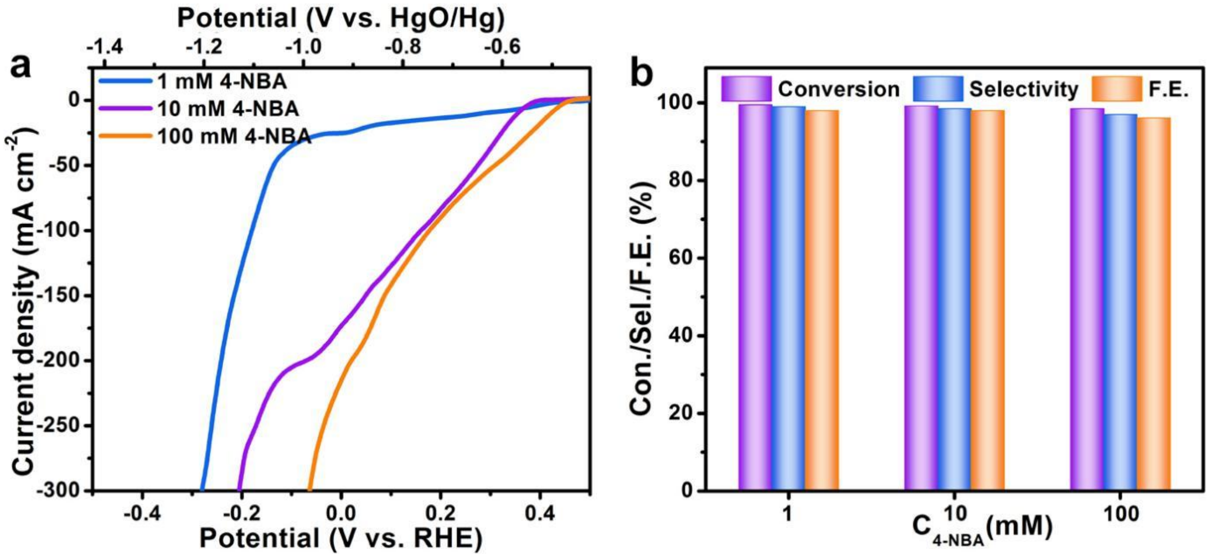

Supplementary Fig. 15 | (a) LSV curves of FeP-MoP/FF for 4-NBA reduction with 1, 10 and 100 mM 4-NBA and (b) Comparison of 4-NBA conversion, 4-ABA selectivity and FE of FeP-MoP/FF for 4-NBA ERR with 1, 10 and 100 mM 4-NBA.

FeP-MoP/FF displays superior catalytic 4-NBA ERR activity with excellent conversion, selectivity and FE at the concentration range of 1~100 mM.

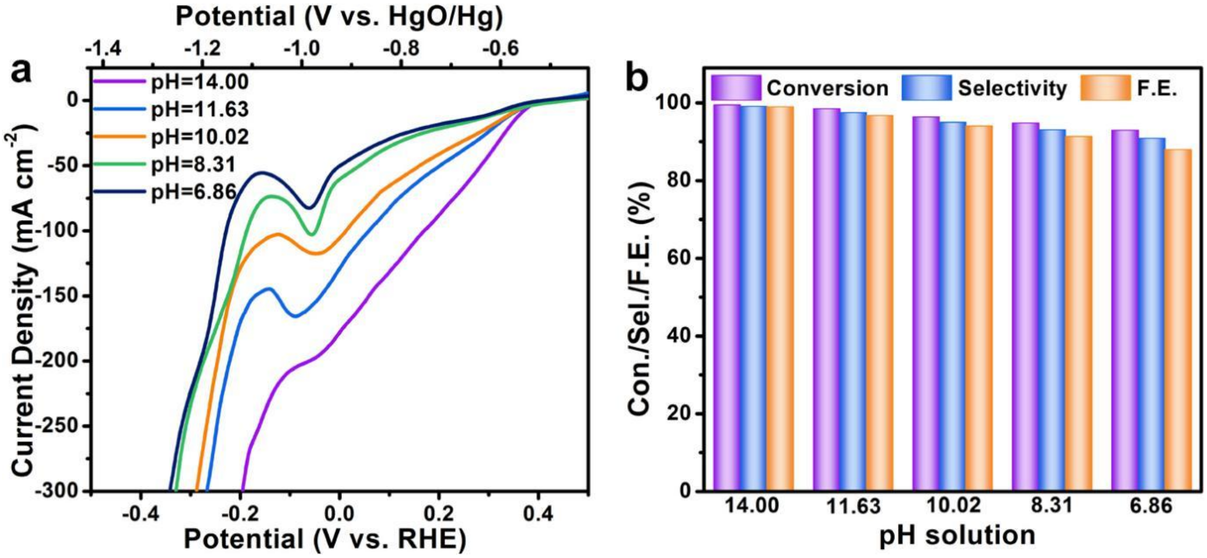

Supplementary Fig. 16 | (a) LSV curves of the 4-NBA ERR in different pH solution. (b) Comparison of 4-NBA conversion, 4-ABA selectivity and FE of FeP-MoP/FF for 4-NBA ERR in different pH solution.

FeP-MoP/FF also shows superb catalytic 4-NBA ERR activity in the neutral and weak alkaline solutions at the pH of 6.86 8.31, 10.02 and 11.63, demonstrating that FeP-MoP/FF can be applied in a wide pH range for 4-NBA ERR

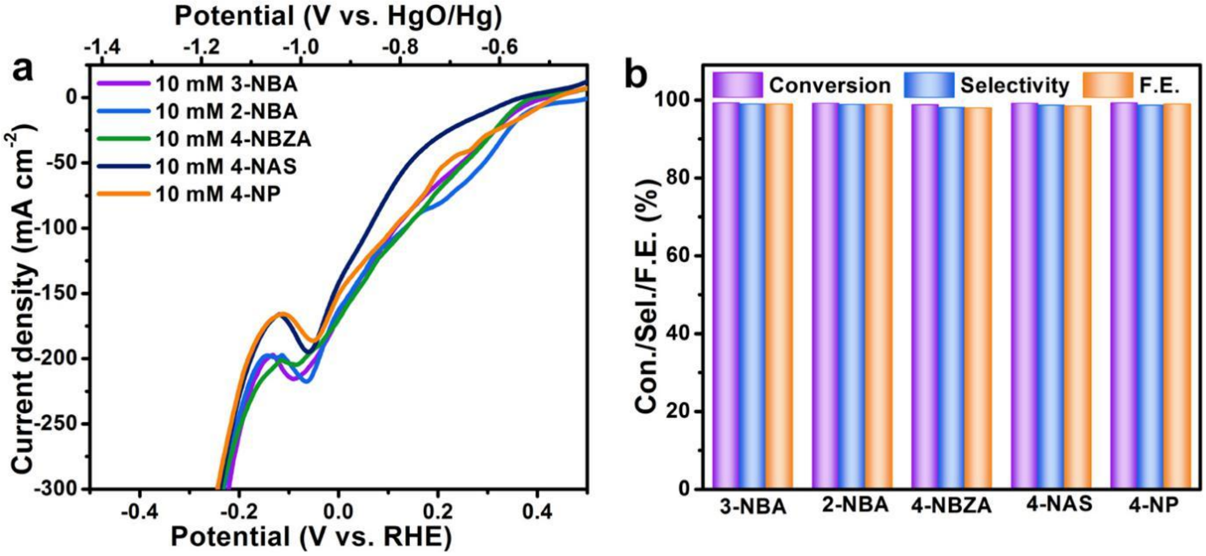

Supplementary Fig. 17 | (a) LSV curves of the electroreduction of 3-NBA, 2-NBA, 4-NBZA, 4-NAS, and 4-NP. (b) Comparison of the conversion, selectivity and FEs of the electroreduction of 3-NBA, 2-NBA, 4-NBZA, 4-NAS, and 4-NP.

Substitution of 4-NBA with other nitrobeneze compounds including 3-NBA, 2-NBA, 4-NAS, 4-NBZA and 4-NP, FeP-MoP/FF still performs well as the electroreduction catalyst with high conversion, selectivity, and FE of these organics.

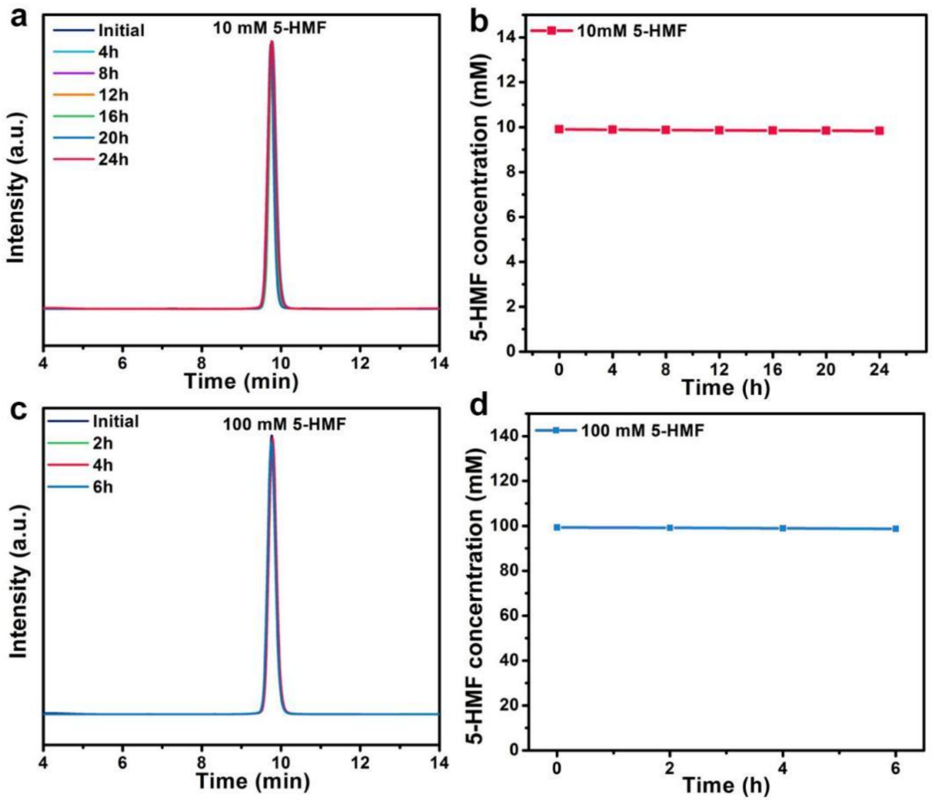

Supplementary Fig. 18 | (a) HPLC-trace acquired at various times and (b) the corresponding stability test of 5-HMF in 1.0 M KOH containing 10 mM 5-HMF under string at the temperature of 298K for 24 h. (c) HPLC-trace acquired at various times and (d) the corresponding stability test of 5-HMF in 1.0 M KOH containing 100 mM 5-HMF under string at the temperature of 298K for 6 h.

We have investigated the stability of HMF in strong alkaline solutions. As shown, there is no side products and the 5-HMF concentration change in 1.0 M KOH solution (pH 14) containing 10 and 100 mM 5-HMF at the temperature of 298K (test conditions in our experiment) for a long time with no adding catalysts, suggesting that 5-HMF is stable in strong alkaline solution without the degradation of HMF or Cannizzaro reaction.

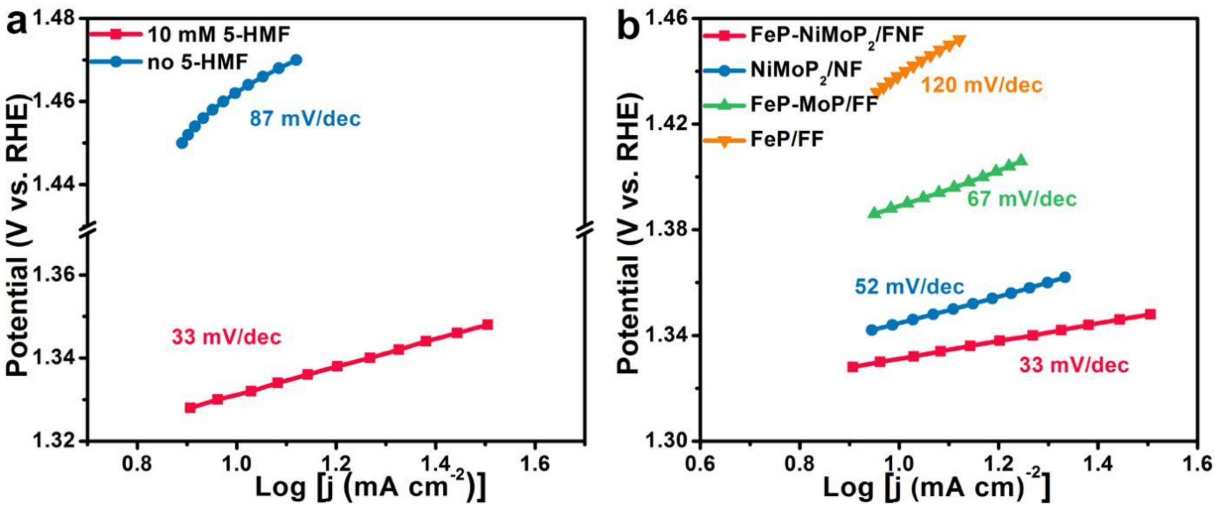

Supplementary Fig. 19 | Tafel slopes of (a) FeP-NiMoP$_2$/FNF in 1.0 M KOH without and with 10 mM 5-HMF, and (b) FeP-NiMoP$_2$/FNF, NiMoP$_2$/NF, FeP-MoP/FF and FeP/FF in 1.0 M KOH with 10 mM 5-HMF.

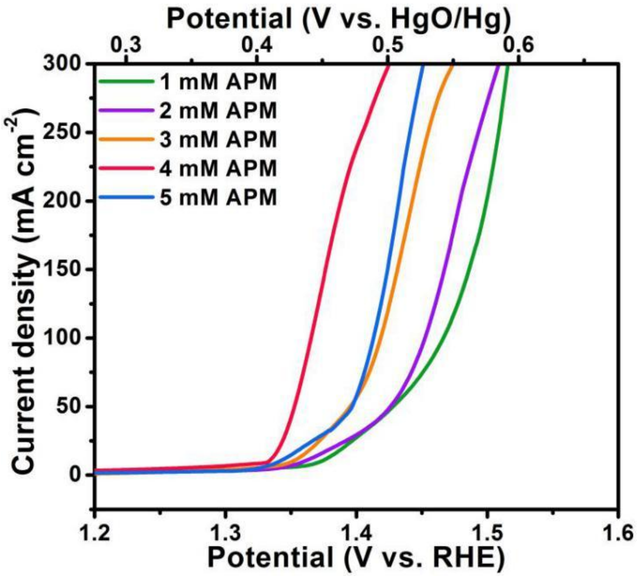

Supplementary Fig. 20 | LSV curves of synthesized FeP-NiMoP_2/FNF by using different APM concentrations for 5-HMF EOR.

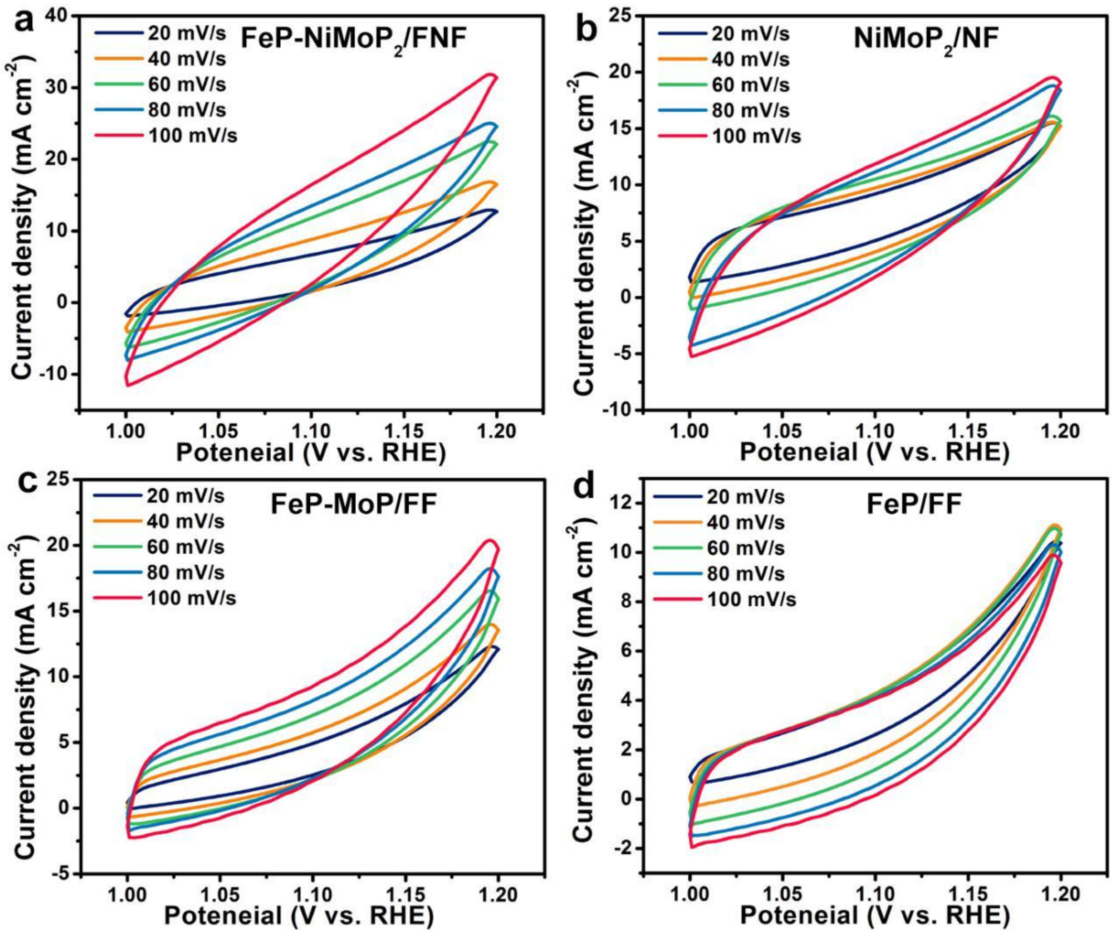

Supplementary Fig. 21 | The capacative CVs of the catalysts for anodic electrooxidation in the region of 1-1.2 V, (a) FeP-NiMoP$_2$/FNF, (b) NiMoP$_2$/NF, (c) FeP-MoP/FF and (d) FeP/FF.

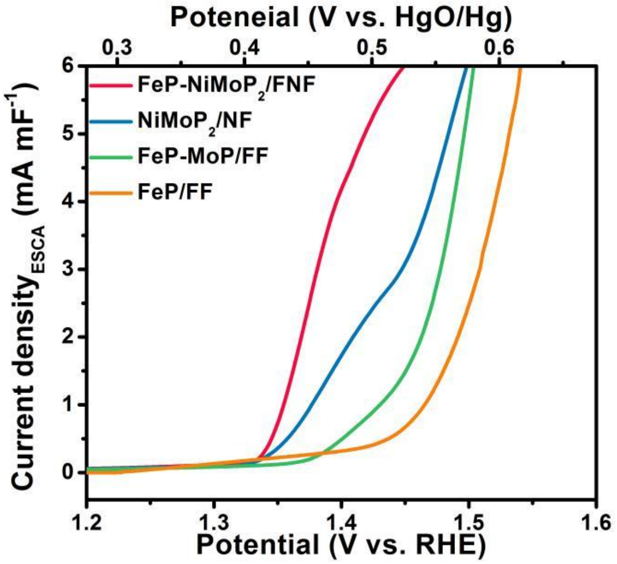

Supplementary Fig 22. Polarization curves with normalization by ECSA of FeP-NiMoP_2/FNF, NiMoP_2/NF, FeP-MoP/FF and FeP/FF

The ECSA-normalized activity of catalysts for 5-HMF EOR are provided. The activity of normalization by ESCA of FeP-NiMoP_2/FNF is also higher than the NiMoP_2/NF, FeP-MoP/FF and FeP/FF. Similarly, FeP-NiMoP_2/FNF also delivers higher activity compared to the FeP/FF and NiMoP_2/FNF, further suggesting the superb intrinsic 5-HMF EOR activity of FeP-NiMoP_2/FNF heterojunction.

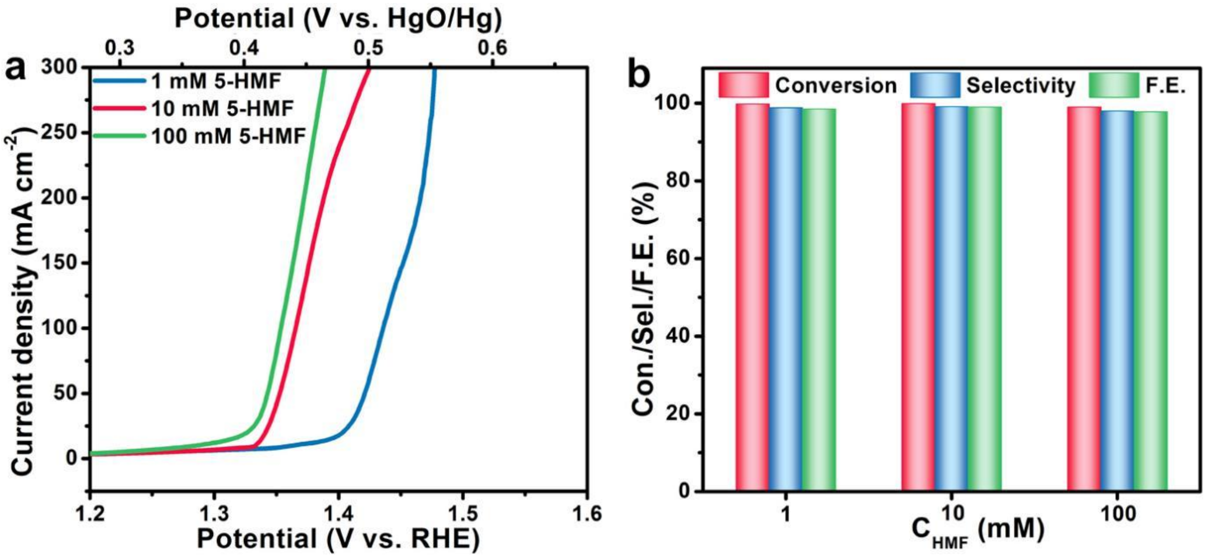

Supplementary Fig. 23 | (a) LSV curves for 5-HMF oxidation with 1, 10 and 100 mM 5-HMF. (b) Comparison of 5-HMF conversion, FDCA selectivity and FE of FeP-NiMoP_2/FNF for 5-HMF EOR with 1, 10 and 100 mM 5-HMF.

In order to also exhibit the practicability of FeP-NiMoP_2/FNF for EOR of biomass, the control experiments similar to the biomass EOR were performed. FeP-NiMoP_2/FNF displays superior 5-HMF EOR activities and excellent 5-HMF conversion ability under different 5-HMF concentrations.

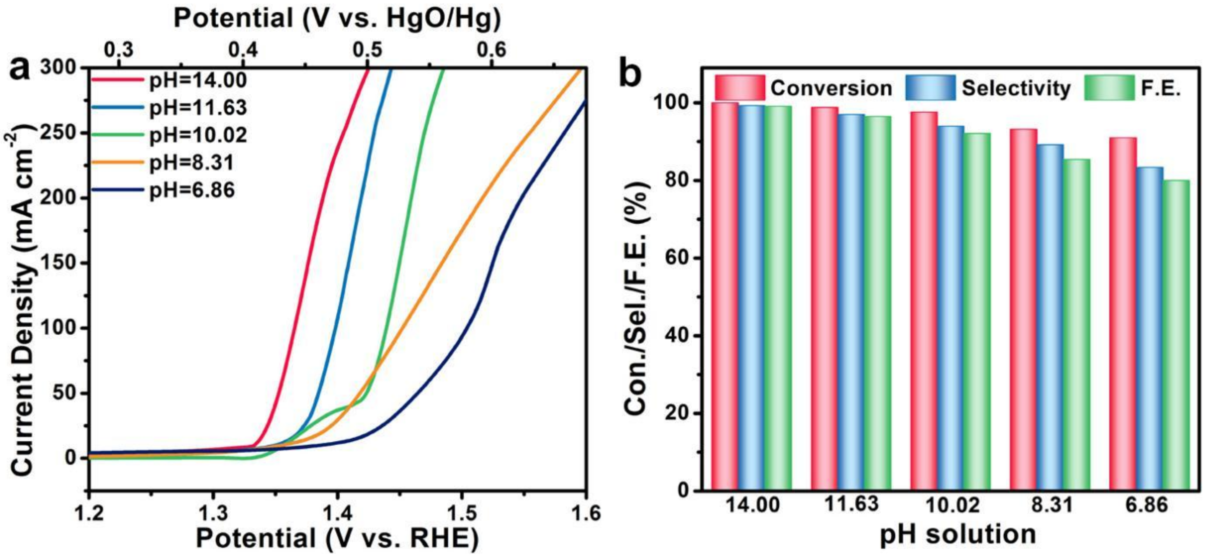

Supplementary Fig. 24 | (a) LSV curves of the 5-HMF EOR in different pH solution. (b) Comparison of 5-HMF conversion, 2,5-FDCA selectivity and FE of FeP-NiMoP_2/FNF for 5-HMF EOR in different pH solution.

Liking the electroreduction side, the electrooxidation reaction also drive smoothly and obtain considerable HMF conversion, selectivity and FE at the neutral and weakly alkaline media.

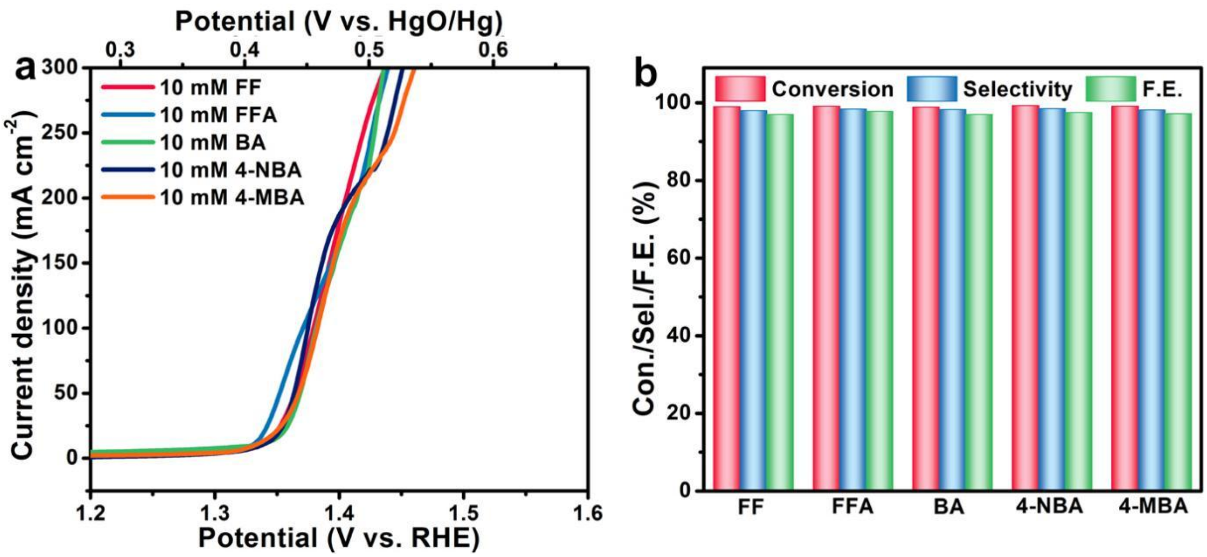

Supplementary Fig. 25 | (a) LSV curves of the electrooxidation of FF, BA, FFA, 4-NBA, and 4-MBA. (b) Comparison of the conversion, selectivity and FE of the electrooxidation of FF, FFA, BA, 4-NBA, and 4-MBA.

When 5-HMF is changed to other biomass organics, including BA, FFA, FFA, 4-NBA, and 4-MBA, FeP-NiMoP_2/FNF still exhibits superior EOR activities. The conversion, selectivity, and FE of these organics substrates exceed 99.0%, 98.0%, and 97.8%, respectively.

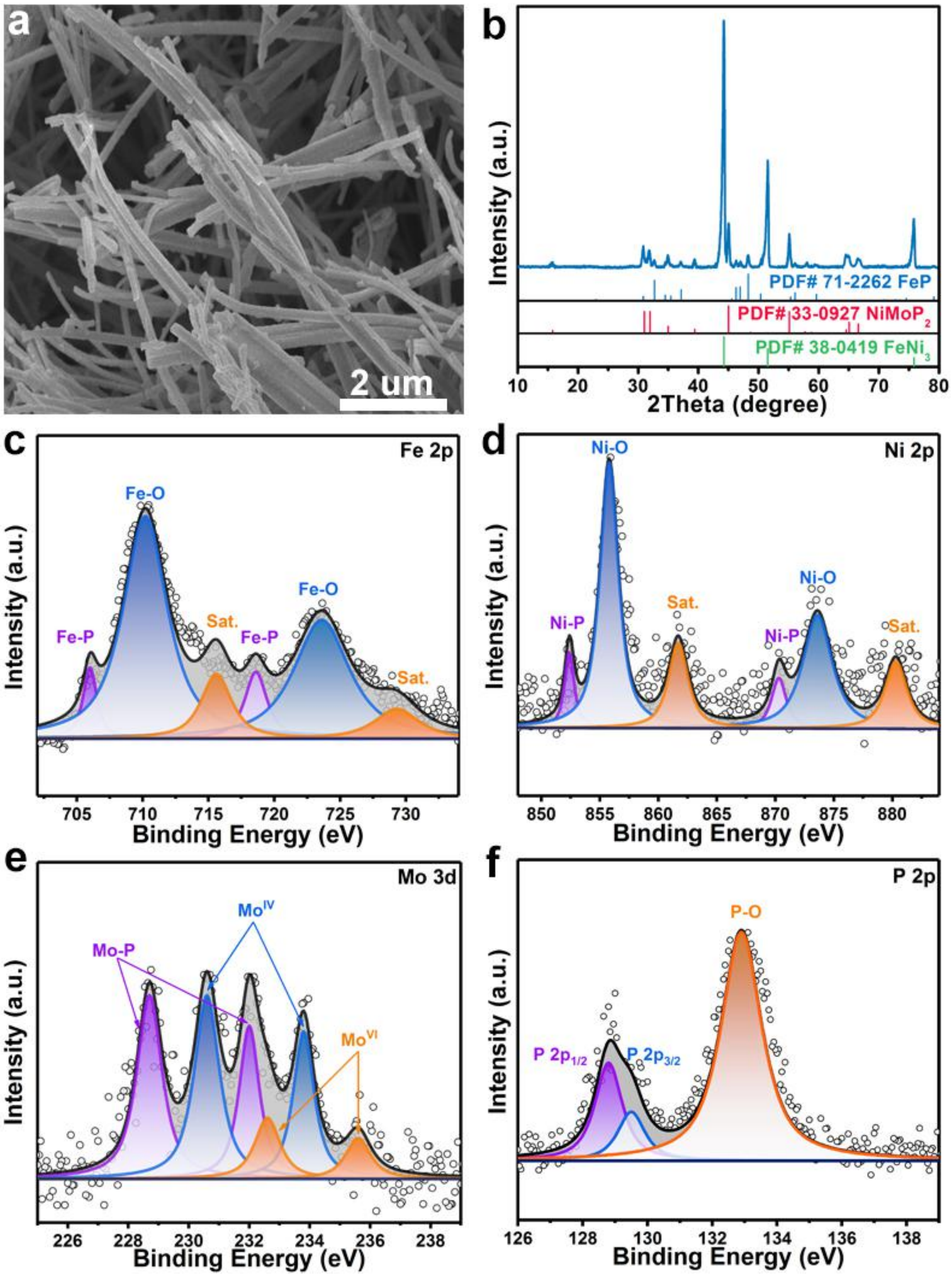

Supplementary Fig. 26 | (a) SEM image, (b) XRD pattern and XPS spectra of (c) Fe 2p, (d) Mo 3d, (e) Ni 2p and (f) P 2p of FeP-NiMoP$_2$/FNF hybrid after 5-HMF EOR for ten successful cycles.

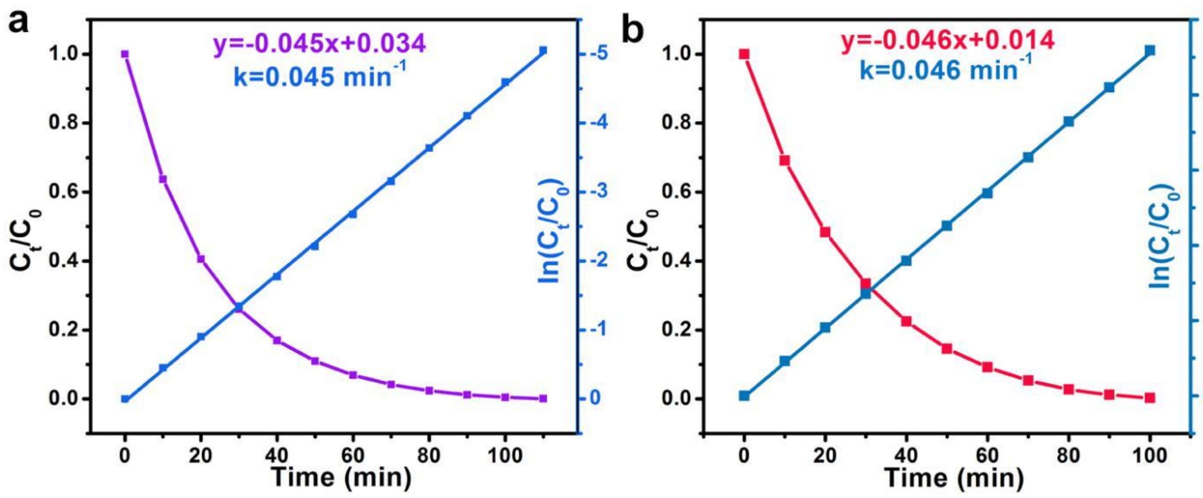

Supplementary Fig. 27 | The dynamic curves of C_t/C_0 and ln(C/C_0) as a function of reaction time for 4-NBA ERR catalyzed by FeP-MoP/FF and 5-HMF EOR catalyzed by FeP-NiMoP_2/FNF.

When the same amount of 4-NBA and 5-HMF were reduced and oxidized, the passing charge were completely at a same amount, The reaction rate of 4-NBA ERR and 5-HMF EOR calculated from the measurement of the reaction kinetics is similar, and thus the adaptation of coupling the 4-NBA ERR and 5-HMF EOR is accessible.

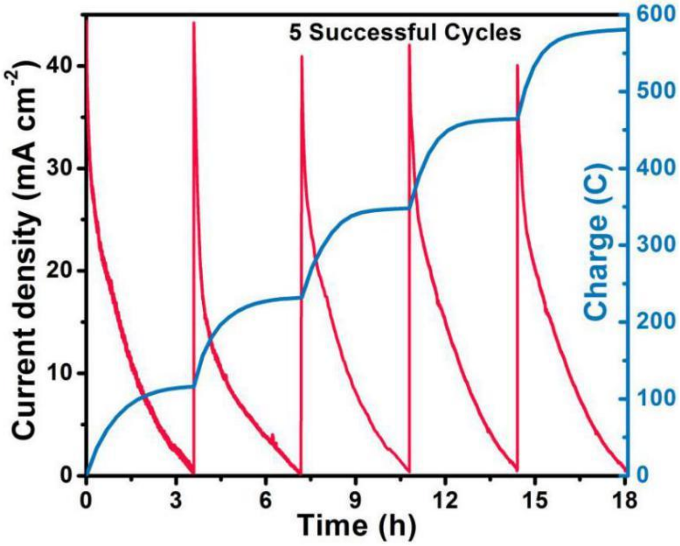

Supplementary Fig. 28 | Current density and and accumulated charge over the time for paired electrocatalysis under five successive cycles.

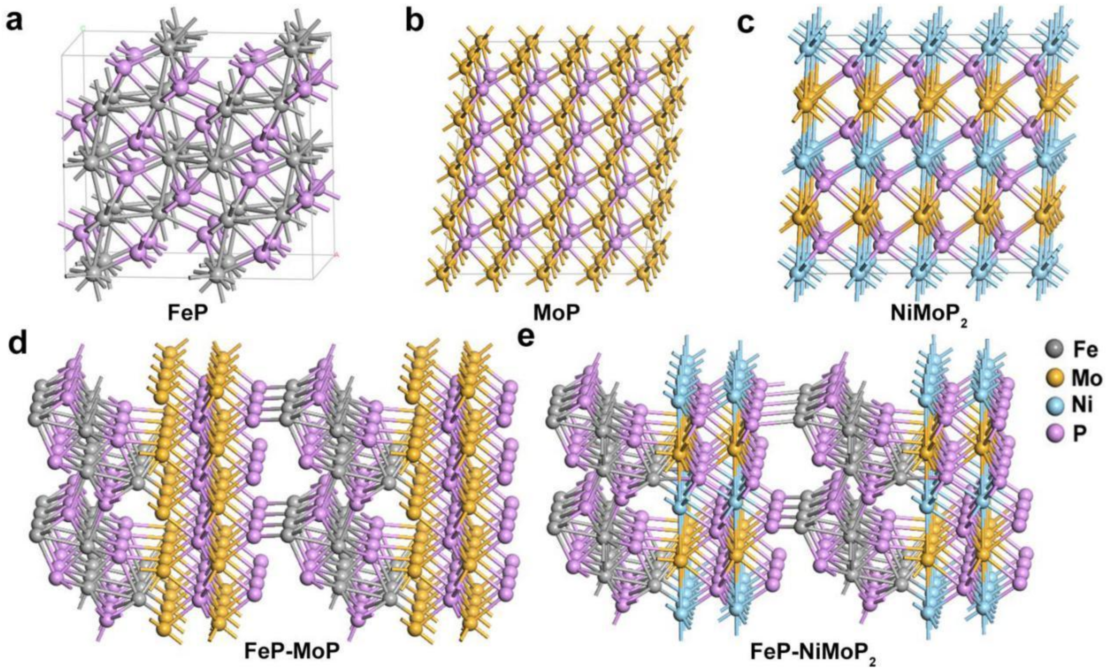

Supplementary Fig. 29 | Theoretical models of (a) FeP, (b) MoP, (c) $NiMoP_2$, (d) FeP-MoP and (e) FeP-$NiMoP_2$.

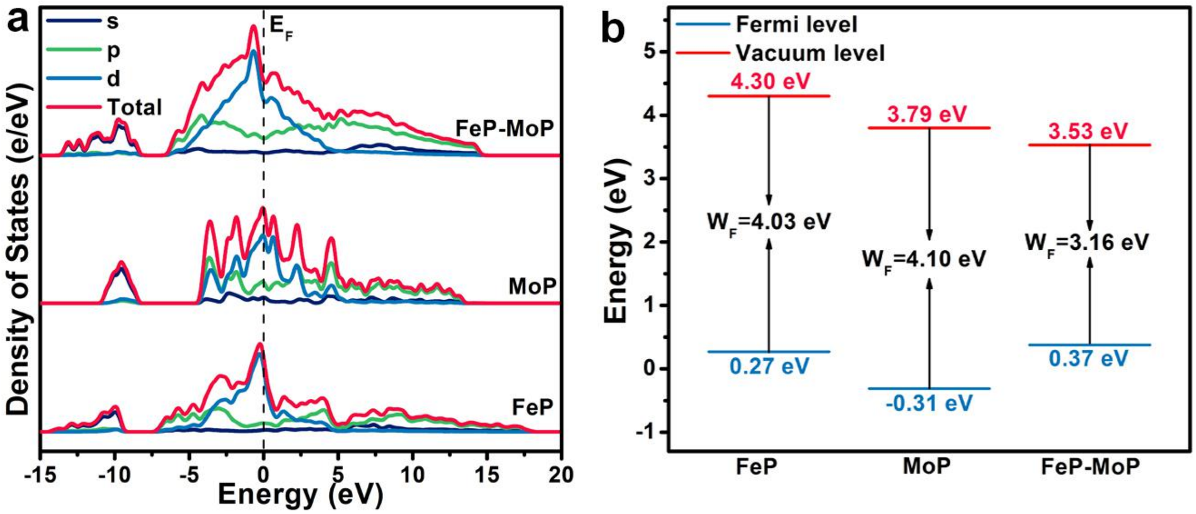

Supplementary Fig. 30 | (a) DOS and (b) Fermi level, Vacuum level and surface work function of FeP, MoP and FeP-MoP.

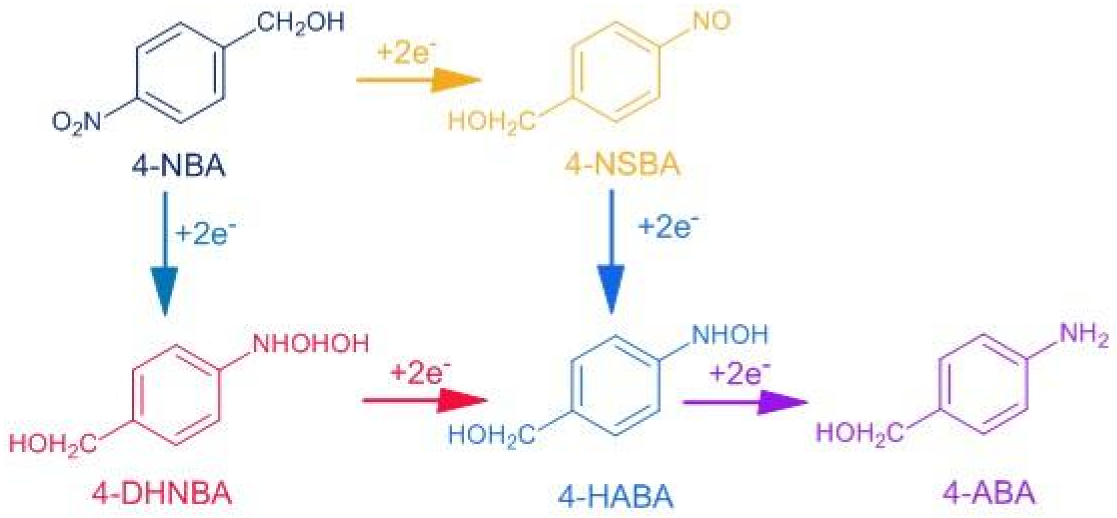

Supplementary Fig. 31 | The possible electroreduction pathway of 4-NBA to 4-ABA

The first step of the 4-NBA reduction pathways were 4-NBA converts to 4-nitrosobenzyl alcohol (4-NSBA) and to the metastable 4-hydrogenatednitrobenzyl alcohol (4-DHNBA). Further reduction of these two intermediate convert into 4-hydroaminobenzyl alcohol (4-HABA), respectively.

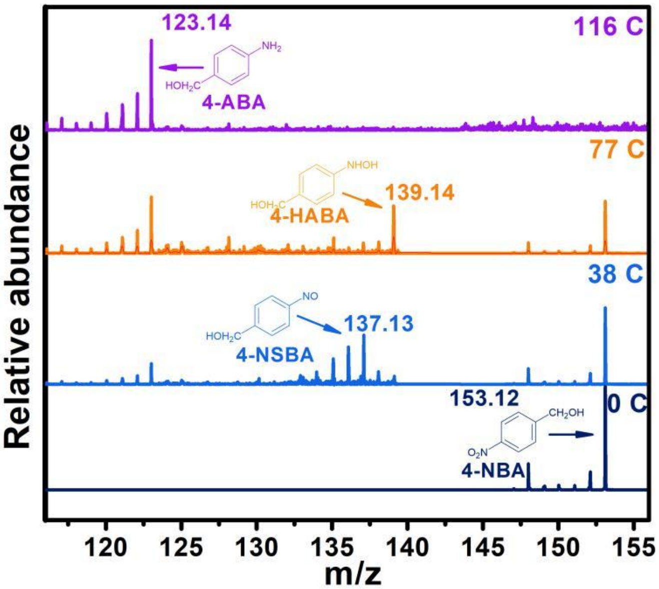

Supplementary Fig. 32 | EMS spectra of reduction of 4-NBA during different passing charge.

To further prove that reaction path over FeP-MoP follows the 4-NSBA path, electrospray mass spectrometry (EMS) was used to analyze the 4-NBA reduction intermediates (4-NSBA and 4-HABA) and final product (4-ABA) during electrolysis. The ESI-MS detected products were analyzed when the charge amounts reached 0, 38, 77 and 116 C, respectively. As shown, the downward peaks at the m/z of 153.12 ($C_7H_7O_3N$) represent the consumption of 4-NBA and the upward peaks at the m/z of 123.14 (C_7H_9ON) represent the formation of 4-ABA. The peaks at the m/z of 137.13 ($C_7H_7O_2N$) are identified to 4-NSBA. The peaks at the m/z of 139.14 (C7H_9O_2N) are index to 4-HABA. The changes of peaks also support that the conversion of 4-NBA into 4-ABA follows the 4-NSBA path.

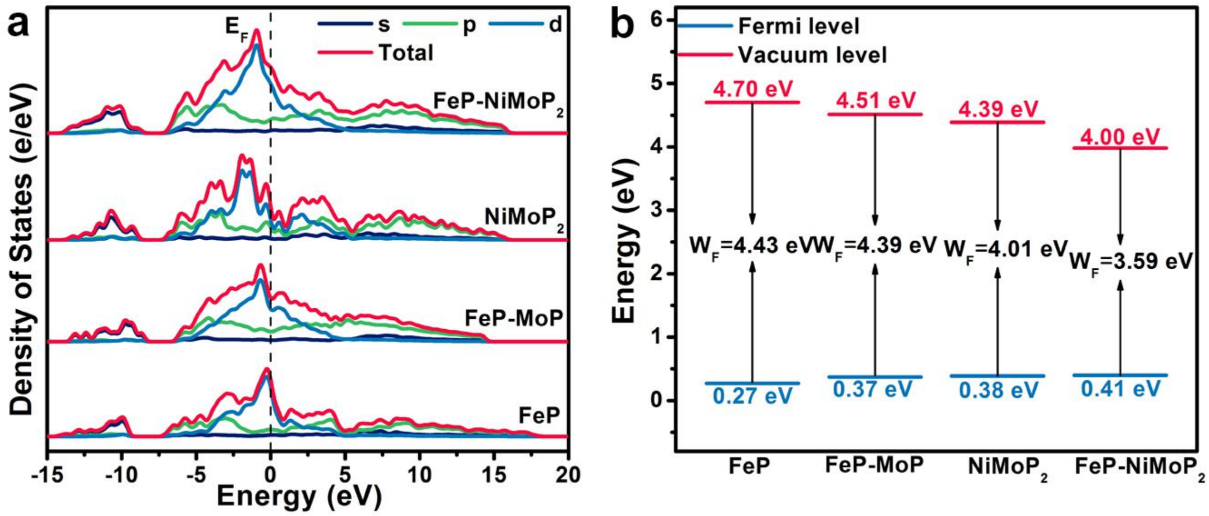

Supplementary Fig. 33 | (a) DOS and (b) Fermi level, Vacuum level and surface work function (W_F) of FeP, FeP-MoP, $NiMoP_2$, and FeP-$NiMoP_2$

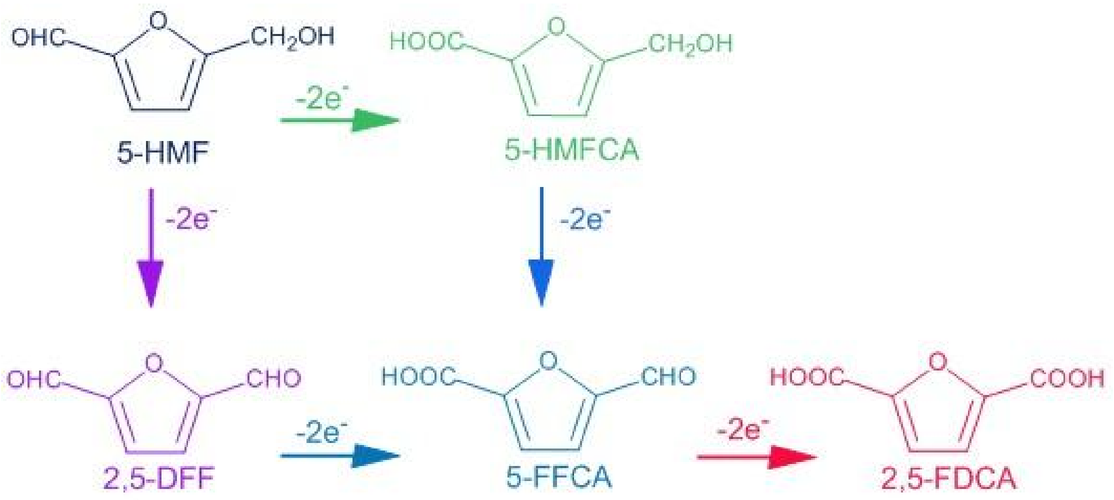

Supplementary Fig. 34 | The possible electrooxidation pathway of 5-HMF to 2,5-FDCA.

The first step of the 5-HMF oxidation pathways is that HMF converts to 5-hydroxymethyl furancarboxylic acid (5-HMFCA) or 2,5-diformylfuran (2,5-DFF). Further oxidation of the 2,5-DFF and 5-HMFCA convert into 5-formylfurancarboxylic acid (5-FFCA), then the 5-FFCA can be convert into and 2,5-furandicarboxylic acid (2,5-FDCA), respectively.

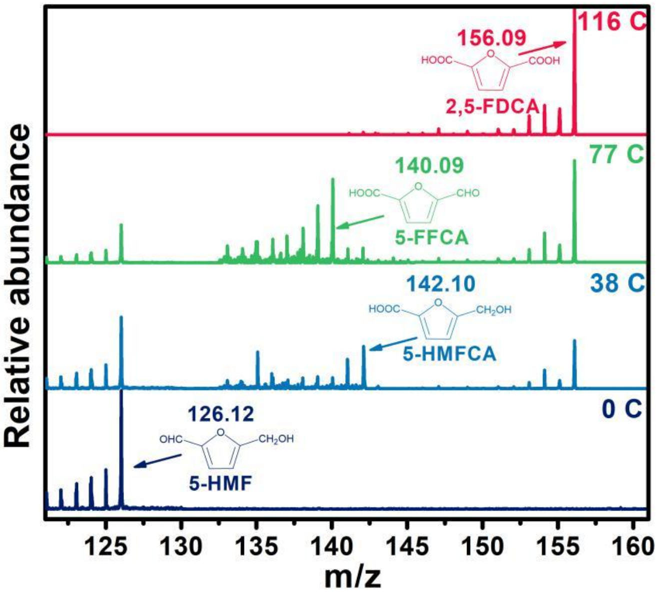

Supplementary Fig. 35 | The EMS spectra of oxidation of 5-HMF during different passing charge.

The EMS technique is also used for the product analysis in the electrooxidation process. The downward peaks at the m/z of 126.10 ($C_6H_6O_3$) represent the consumption of 5-HMF and he upward peaks at the m/z of 156.09 represent the formation of 2,5-FDCA ($C_6H_4O_5$). The peak at the m/z of 142.10, corresponding to the carboxylic anion of 5-HMFCA ($C_6H_6O_4$). The peaks occurred at the m/z 140.09, which can correspond with the 5-FFCA ($C_6H_4O_4$). The changes of peaks further validate the oxidation of 5-HMF follows the 5-HMFCA path.

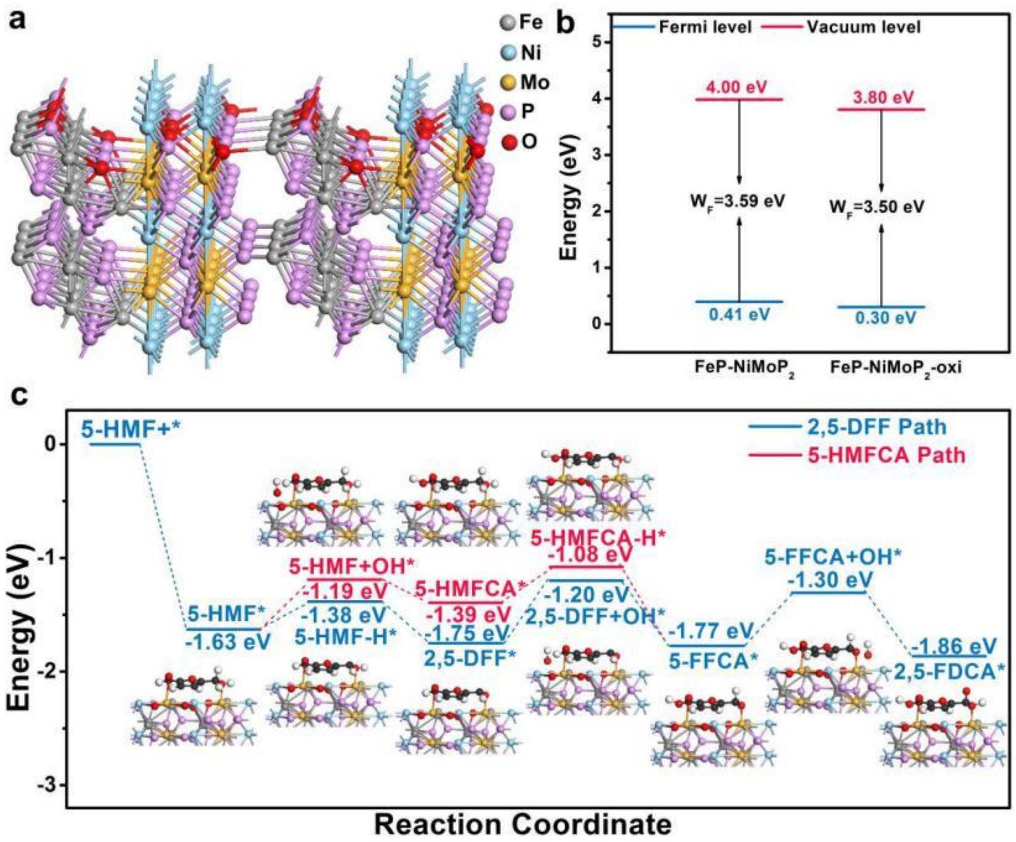

Supplementary Fig. 36 | (a) Theoretical model of FeP-NiMoP$_2$-oxi).(b) The calculated working function of FeP-NiMoP$_2$ and FeP-NiMoP$_2$-oxi). (c) Energy profiles of 5-HMF oxidation to 2,5-FDCA on the FeP-NiMoP$_2$-oxi) surface.

2. Supplementary Figures

Supplementary Table 1 | The 4-NBA EHR performance of the different catalysts.

Parameters	FeP-MoP/FF	FeP/FF	MoP/FF
**η_{10} (V)**	0.364	0.337	0.319
**η_{100} (V)**	0.169	0.051	-0.149
**Tafel slopes (mV dec^{-1})**	38	64	82
**Conversion (%)**	99.5	96.5	92.0
**Selectivity (%)**	99.1	95.5	90.2
**FE (%)**	99.0	94.0	88.0

Supplementary Table 2 | Comparison of conversion, selectivity and FE of FeP-MoP/FF with the reported catalysts that catalyzed electroreduction of nitro compounds.

Samples	Organics	Products	Con. (%)	Sel. (%)	F.E. (%)	Ref.
CoP/NF	Nitrobenzene	aniline	99.0	99.0	98.0	[1]
CoP/NF	4-NAS	4-anisidine	98.0	98.5	98.0	[1]
CoP/NF	4-NP	4-aminophenol	96.0	98.5	98.0	[1]
FeP/NF	Nitrobenzene	aniline	95.0	90.0	N/A	[1]
Ni_2P/NF	Nitrobenzene	aniline	75.0	70.0	N/A	[1]
Co_3S_{4-x}/Ti	Nitrobenzene	aniline	98.0	99.0	98.0	[2]
Co_3S_{4-x}/Ti	4-NAS	4-anisidine	96.0	98.0	96.0	[2]
Co_3S_{4-x}/Ti	4-NP	4-aminophenol	99.0	98.0	96.0	[2]
NixB/NF	4-NP	4-aminophenol	99.0	99.0	99.0	[3]
Pd/C	4-NAS	4-anisidine	90.0	87.0	N/A	[4]
Pd/C	Nitrobenzene	aniline	98.0	95.0	N/A	[4]
FeP-MoP/FF	4-NBA	4-ABA	99.4	99.1	99.0	This work
FeP-MoP/FF	4-NAS	4-anisidine	99.2	98.8	99.0	This work
FeP-MoP/FF	4-NP	4-aminophenol	99.0	98.5	98.5	This work

Supplementary Table 3 | Electroreduction of various organic substrates by using FeP-MoP/FF as catalyst at the potential of 0.004 V

**Biomass substrates**	**Products**	**η_{10} (V)**	**Con. (%)**	**Sel. (%)**	**F.E. (%)**
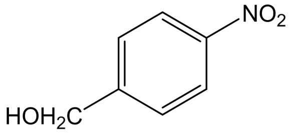	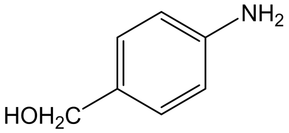	0.364	99.5	99.1	99.0
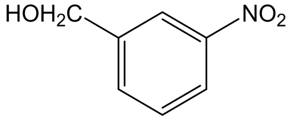	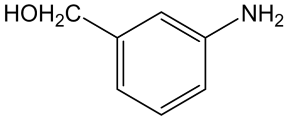	0.361	99.1	98.6	98.7
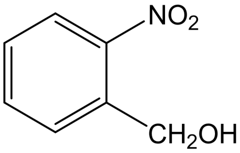	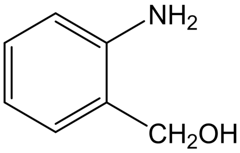	0.360	99.0	98.5	98.5
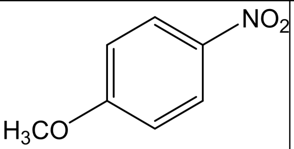	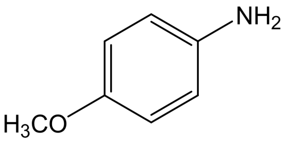	0.358	99.2	98.8	99.0
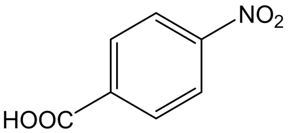	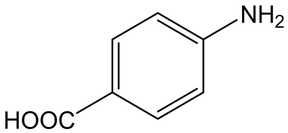	0.355	98.7	98.0	98.0
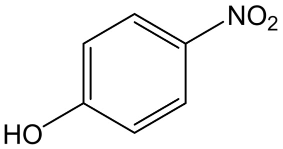	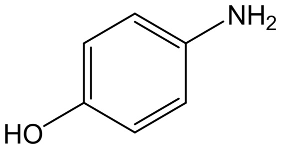	0.361	99.3	98.7	99.0

Supplementary Table 4 | The 5-HMF EOR performance of the different catalysts.

Parameters	FeP-NiMoP$_2$/FNF	NiMoP$_2$/NF	FeP-MoP/ FF	FeP/FF
η_{10} (V)	1.333	1.345	1.387	1.425
η_{100} (V)	1.366	1.434	1.490	1.504
Tafel slopes (mV dec^{-1})	33	52	67	120
Conversion (%)	100	99.0	98.0	90.5
Selectivity (%)	99.2	98.2	97.4	88.2
FE (%)	99	98.0	97.0	85.0

Supplementary Table 5 | Comparison of conversion, selectivity and FE of FeP-$NiMoP_2$/FNF with reported catalysts that catalyzed electrooxidation of various organics.

**Samples**	**Organics**	**Products**	**Con. (%)**	**Sel. (%)**	**F.E. (%)**	**Ref.**
NixB/NF	5-HMF	2,5-FDCA	~100	99.0	99.0	[3]
Ni_3S_2/NF	5-HMF	2,5-FDCA	~100	99.0	98.0	[5]
Ni_3S_2/NF	BA	BZA	99.0	98.0	98.0	[5]
Ni_3S_2/NF	FF	FFA	99.0	97.0	97.0	[5]
Co-P/CF	5-HMF	2,5-FDCA	99.0	>90.0	>90.0	[6]
Ni_2P/NPA /NF	5-HMF	2,5-FDCA	~100	98.0	98.0	[7]
Ni_2P/CFP	FF	FA	99.0	98.5	98.0	[8]
Ni_3N@C/NF	5-HMF	2,5-FDCA	99.5	98.5	98.5	[9]
MoO_2-FeP @C	5-HMF	2,5-FDCA	99.4	98.6	97.8	[10]
hp-Ni/NF	5-HMF	2,5-FDCA	98.0	97.0	97.0	[11]
hp-Ni/NF	BA	BZA	99.0	98.0	97.0	[11]
FeP-$NiMoP_2$ /FNF	5-HMF	2,5-FDCA	~100	99.2	99.0	This work
FeP-$NiMoP_2$ /FNF	BA	BZA	99.4	98.7	98.7	This work
FeP-$NiMoP_2$ /FNF	FF	FA	99.7	99.0	99.0	This work

Supplementary Table 6 | Electrooxidation of various biomass substrates by using FeP-$NiMoP_2$/FNF as a catalyst at the potential of 1.404 V

Biomass substrates	Products	η_{10} (V)	Con. (%)	Sel. (%)	F.E. (%)
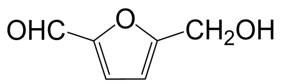	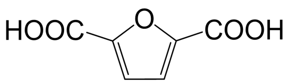	1.333	100	99.2	99.0
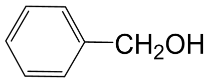	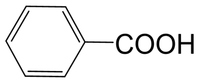	1.341	99.4	98.7	98.7
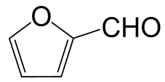	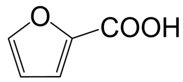	1.341	99.7	99.0	99.0
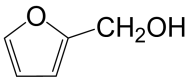	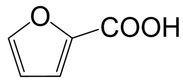	1.340	99.0	98.0	97.8
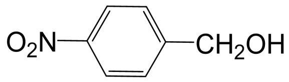	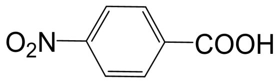	1.346	99.3	98.5	98.4
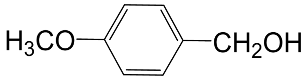	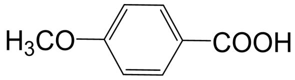	1.345	99.2	98.3	98.0

Supplementary Table 7 | XPS and ICP-OES analyses of FeP-NiMoP$_2$/FNF before and after 5-HMF EOR cyclic stability test.

Element		Fe	Ni	Mo	P
XPS (wt.%)	Before 5-HMF EOR	25.21	32.32	30.02	12.45
	After 5-HMF EOR	25.98	32.91	30.63	10.48
ICP-OES (μg mL^{-1})	The content in solution after 5-HMF EOR	0.1	0.1	0.4	5.6

Supplementary Table 8 | The adsorption energies of 4-NBA on different sites of FeP-MoP, FeP-$NiMoP_2$, FeP and MoP surfaces.

<table>
<tr><th>Models</th><th>FeP-MoP</th><th>FeP-$NiMoP_2$</th><th>FeP</th><th>MoP</th></tr>
<tr><td rowspan="5">Energy (eV)</td><td>-1.33 (Fe site)</td><td>-1.29 (Fe site)</td><td rowspan="2">-1.15(Fe site)</td><td rowspan="2">-0.82 (Mo site)</td></tr>
<tr><td>-1.08 (Mo site)</td><td>-1.12 (Mo site)</td></tr>
<tr><td>-0.91 (P-FeP site)</td><td>-0.96 (Ni site)</td><td rowspan="3">-0.79 (P site)</td><td rowspan="3">-0.68 (P site)</td></tr>
<tr><td rowspan="2">-0.81 (P-MoP site)</td><td>-0.90 (P-FeP site)</td></tr>
<tr><td>-0.75 (P-$NiMoP_2$ site)</td></tr>
</table>

Supplementary Table 9 | The adsorption energies of 5-HMF on different sites of FeP-$NiMoP_2$, FeP-$NiMoP_2$-oxi, FeP-MoP, $NiMoP_2$ and FeP surfaces.

<table>
<tr><th>Models</th><th>FeP-$NiMoP_2$</th><th>FeP-$NiMoP_2$-oxi</th><th>FeP-MoP</th><th>$NiMoP_2$</th><th>FeP</th></tr>
<tr><td rowspan="7">Energy (eV)</td><td>-1.60 (Mo site)</td><td>-1.63 (Mo site)</td><td rowspan="2">-1.15 (Mo site)</td><td rowspan="2">-1.32 (Mo site)</td><td rowspan="4">-0.96 (Fe site)</td></tr>
<tr><td>-1.47 (Ni site)</td><td>-1.56 (Ni site)</td></tr>
<tr><td rowspan="2">-1.14 (Fe site)</td><td>-1.12 (Fe site)</td><td rowspan="2">-0.89 (Fe site)</td><td rowspan="3">-1.16 (Ni site)</td></tr>
<tr><td>-1.12 (O-$NiMoP_2$-oxi site)</td></tr>
<tr><td rowspan="2">-0.96 (P-$NiMoP_2$ site)</td><td>-0.97 (O-FeP-oxi site)</td><td rowspan="2">-0.75 (P-FeP site)</td><td rowspan="3">-0.71 (P site)</td></tr>
<tr><td>-0.92 (P-$NiMoP_2$-oxi site)</td><td rowspan="2">-0.90 (P site)</td></tr>
<tr><td>-0.88 (P-FeP site)</td><td>-0.87 (P-FeP-oxi site)</td><td>-0.62 (P-MoPsite)</td></tr>
</table>

Reference

1. Zhang, B., Huang, C., Huang, Y., Liu, C. & Chong, X. Potential-tuned selective electrosynthesis of azoxy-, azo- and amino-aromatics over a CoP nanosheet cathode. *Nati. Sci. Rev.* **7**, 285-295 (2020).
2. Zhao, Y., Liu, C., Wang, C., Chong, X. & Zhang B. Sulfur vacancy-promoted highly selective electrosynthesis of functionalized aminoarenes via transfer hydrogenation of nitroarenes with H_2O over a Co_3S_{4-x} nanosheet cathode. *CCS Chem.* **7**, 507-515 (2020).
3. Zhang, P. et al. Paired electrocatalytic oxygenation and hydrogenation of organic substrates with water as the oxygen and hydrogen source. *Angew. Chem. Int. Ed.* **58**, 9155-9159 (2019).
4. Gevorgyan, A., Mkrtchyan, S., Grigoryan, T. & Iaroshenko, V. O. application of silicon-initiated water splitting for the reduction of organic substrates. *ChemPlusChem* **83**, 375-382 (2018).
5. You, B., Liu, X., Jiang, N. & Sun, Y. A general strategy for decoupled hydrogen production from water splitting by integrating oxidative biomass valorization. *J. Am. Chem. Soc.* **138**, 13639-13646 (2016).
6. Jiang, N., You, B., Boonstra, R., Terrero, R. & Sun, Y. Integrating electrocatalytic 5-hydroxymethylfurfural oxidation and hydrogen production via Co-P derived electrocatalysts. *ACS Energy Lett.* **1**, 386-390 (2016).
7. You, B., Jiang, N., Liu, X. & Sun, Y. Simultaneous H_2 generation and biomass upgrading in water by an efficient noble-metal-free bifunctional electrocatalyst. *Angew. Chem. Int. Ed.* **55**, 9913-9917 (2016).
8. Zhang, X. et al. Simultaneously high-rate furfural hydrogenation and oxidation upgrading on nanostructured transition metal phosphides through electrocatalytic conversion at ambient conditions. *Appl. Catal. B. Environ.* **244**, 899-908 (2019).
9. Zhang, N. et al. Electrochemical oxidation of 5-hydroxymethylfurfural on nickel nitride/carbon nanosheets: Reaction pathway determined by in situ sum frequency generation vibrational spectroscopy. *Angew. Chem. Int. Ed.* **58**, 15895-15903 (2019).
10. Yang, G. et al. Interfacial engineering of MoO_2-FeP heterojunction for highly efficient hydrogen evolution coupled with biomass electrooxidation. *Adv. Mater.* **32**, 2000455 (2020).

11. You, B., Liu, X., Liu, X. & Sun, Y. Efficient H_2 evolution coupled with oxidative refining of alcohols via a hierarchically porous nickel bifunctional electrocatalyst. *ACS Catal.* **7**, 4564-4570 (2017).
